# Determinants of intention to use family planning methods in the four emerging regions of Ethiopia: an ideation score based assessment

**DOI:** 10.1186/s12978-022-01385-y

**Published:** 2022-06-13

**Authors:** Tewodros Getinet, Feiruz Surur, Balkachew Nigatu, Alula Meressa, Yonas Abesha, Munir Kassa, Merhawi Gebremedhin, Delayehu Bekele

**Affiliations:** 1 School of Public Health, St Paul’s Hospitals Millennium Medical College, Addis Ababa, Ethiopia; 2 Department of Obstetrics and Gynecology, St Paul’s Hospitals Millennium Medical College, Addis Ababa, Ethiopia; 3 Research Advisor, St Paul’s Hospitals Millennium Medical College, Addis Ababa, Ethiopia; 4 Individual Consultant, Addis Ababa, Ethiopia; 5 Federal Ministry of Health, Addis Ababa, Ethiopia; 6 School of Public Health, Haramaya University, Harar, Ethiopia

**Keywords:** Ideation, Confirmatory factor analysis, Family planning, Ethiopia

## Abstract

**Background:**

Ideation refers to the ideas and views that people hold; it has been identified as an important explanation for differences in contraceptive use within and across countries. This study aimed to identify ideational factors that influence intention to use family planning (FP) methods among women of reproductive age (WRA) in the four emerging regions of Ethiopia.

**Methods:**

A quantitative cross-sectional survey of 2891 WRA was carried out in the four emerging regions of Ethiopia. A multistage, stratified systematic random sampling technique was employed to select the study participants. Data were collected by trained enumerators, using tablets equipped with Open Data Kit. To assess the impact of ideation on intention to use FP, the research team used 41 items distributed across five broad ideational factors: contraception awareness, self-efficacy, rejection of myth and rumor, intra-family discussion and family support. Confirmatory factor analysis was employed to test the fit of these items into the five ideational factors. A multiple binary logistic regression analysis was employed to assess the combined effect of these ideational factors with different sociodemographic variables on intention to use contraceptive methods. In all the statistical analysis, a *p*-value < 0.05 was considered statistically significant.

**Results:**

Different proportions of women in the four regions intended to use contraceptives in the future: 74.9% in Benishangul-Gumuz, 50.1% in Gambela, 21.8% in Afar, and 20.1% in Somali. The proportion of women who intended to use contraceptives varied with ideation scores. The multiple binary logistic regression revealed that self-efficacy was an important ideational factor of intention to use contraception in all four regions. Rejection of myth and rumor was also an important factor in all regions except in Somali. Contraception awareness and family support were significant predictors of intention to use contraception in the Afar region only. Intra-family discussion was not found significant in any region.

**Conclusions:**

Regional/district health offices should focus on increasing self-efficacy for FP use. Demystifying rumors would contribute to improved intention to use FP among women in Afar, Benishangul-Gumuz, and Gambela regions. Raising contraception awareness and encouraging family support would improve intention to use FP in Afar region.

## Plain English summary

Contraceptive use shows sizeable differences within and across Ethiopia, and the situation is worsening in the country’s emerging regions (Afar, Benishangul-Gumuz, Gambela, and Somali). Little or no progress in expanding access to family planning (FP) services in these emerging regions has been observed over the past years. Reasons for the disparity and the psychosocial factors expected to affect uptake of contraceptives are not clearly identified in Ethiopia’s emerging regions.

In this study, we explored intention to use family planning in the future and the impact of ideation on this intention among 2891 women of reproductive age in the four emerging regions of Ethiopia. Ideation is the concept that people’s actions are influenced strongly by their beliefs, ideas, and feelings (‘‘ideational factors’’) and provides a framework to understand behaviors, including contraceptive behavior. This study indicates that women’s intention to use FP in the future increases with increases in their ideational factors: contraception awareness, self-efficacy, rejection of myth and rumor, intra-family discussion and family support. Findings from this study should inspire health officials to focus on the psychosocial factors to close the disparity and improve contraceptive utilization.

## Background

Ethiopia has recorded significant progress in improving its population’s access to family planning (FP). According to the Ethiopian demographic health surveys (EDHS), contraceptive prevalence rate (CPR) has increased from 8% in 2000 to 36% in 2016, a fivefold increase [1–4]. At the same time, unmet need, meaning the proportion of fecund women who are not using contraception but who wish to postpone their next birth or stop childbearing altogether, decreased from 37% in the 2000 EDHS to 22% in 2016 [1–4]. Knowledge about FP methods is near universal, with 98.3% of women surveyed able to identify at least one modern contraceptive method [4]. The country has also massively expanded health care facilities and the Federal Ministry of Health (FMoH) estimated that 90% of the population had access to health care in 2011 [5]. The most recent service provision survey estimated that 87% of health facilities (excluding health posts) offered modern family planning methods [6].

Ethiopia set ambitious targets for the year 2020 to increase the CPR to 55%, reduce the total fertility rate from 4.1 in 2018 to 3, and decrease the unmet need for FP to 10% [7]. Despite overall progress in decreasing in the unmet need for FP, there is substantial inequality in FP access across population groups due to socioeconomic, education and place of residence factors [7]. There are also sizeable cross-regional differences in contraceptive use, and little or no progress in expanding access to FP services in the four emerging regions (Afar, Benishangul-Gumuz, Gambela, and Somali) has been observed over the past years [1–4]. Afar and Somali in particular lag behind national contraceptive prevalence with rates of modern contraceptive use of 12% and 1%, respectively. Contraceptive prevalence in Benishangul-Gumuz (28%) and Gambela (35%) is closer to the national average [4]. Reasons for the disparity and psychosocial factors that expect to affecting uptake of contraceptives are not clearly identified in Ethiopia’s emerging regions.

Globally, factors beyond access to family planning and health services have been shown to affect rates of contraceptive use. These factors may include socioeconomic and cultural determinants, household factors such as partner’s attitude and support, and individual determinants including knowledge and attitudes toward FP.

Ideation provides a lens through which these individual level factors may be explored. Ideation is the concept that people’s actions are influenced strongly by their beliefs, ideas, and feelings (‘‘ideational factors’’); it provides a framework to understand behaviors, including contraceptive behavior [8]. Some studies have posited ideational change to be a key factor in the second demographic transition in Europe [9, 10] and in recent fertility declines in some less industrialized countries [11]. Ideation models have been used among demographers and public health experts to better understand how the individual psychosocial factors may influence family planning use and contraceptive uptake [8, 12–15]. The ideation model conceptualizes and structures the psychosocial factors of behavior, thus enabling a greater understanding of the multiple factors that determine family planning behaviors.

This study used Kincaid’s modified version of ideation model, which links individual ideation with behavior, and the Ideation Model of Strategic Communication with Behavioral Change [16]. This model comprises three domains: cognitive, emotional, and elements of social interactions. The cognitive dimension includes the following psychosocial concepts: attitudes, knowledge, perceived risk, subjective norms, and self-image. The emotional domain includes preferences and self-efficacy. Finally, the social elements of ideation include social support; social influence; interpersonal communication (e.g., spousal communication); and personal advocacy [16–18].

In this paper, using a modified ideation model, we aimed to identify ideational factors that influence intention to use FP methods among married and unmarried women of reproductive age (WRA) in the four emerging regions of Ethiopia.

## Methods

### Study design, setting, and population

A quantitative, cross-sectional survey was conducted among women age 15–49 years living in the four emerging regions of Ethiopia. Benishangul-Gumuz and Gambela are found in the western part of the country, and Afar and Somali are located in the eastern part of the country. These emerging regions are known for having lower population density, less developed infrastructure, and lower coverage of most health services relative to other parts of the country. Afar and Somali regions have a predominantly pastoralist population. These regions have the lowest FP uptake and a declining trend of FP use over the past 20 years [1–4]. Although Benishangul-Gumuz and Gambela have contraceptive use rates that are closer to the national average, little information is known about how these rates differ across urban and rural areas. The study was conducted over a period of five months (December 2017 to April 2018).

### Sample size determination and sampling

The sample size for this survey was estimated separately for each of the four emerging regions, to ensure reliable information from each region, considering their unique sociocultural characteristics. The sample sizes in each region were determined by considering intention to use FP as key outcome indicator. Samples sizes totaled 683 for Afar, 805 for Benishangul-Gumuz, 678 for Somali, and 753 for Gambela.

Once we determined the sample sizes, a multistage random sampling technique was employed to select study participants. For the first stage, we listed all districts for each region and used probability proportional to their size to randomly select 20% of the districts for inclusion in the survey. At the second stage, the survey team used the register of the households in the selected districts from the district administration and stratified the households by rural/urban location. Household samples were proportionally allocated according to strata size and selected using systematic random sampling technique. For each randomly selected household, all eligible women were approached; if there was more than one eligible woman, one was randomly selected using a lottery method.

### Study instruments and data collection

We developed a semi structured questionnaire, using questions from the EDHS and other survey tools. To meet the additional objectives of the assessment ideation score on the intention to use FP methods, we convened a panel of experts (the authors plus other professionals in the area of reproductive health), asking them to focus on questions that would assist in determining the ideation score. The study variables include intention to use FP, age, educational status, marital status, place of residence (rural versus urban), occupation, religion, family income, prior use of contraceptive, partner’s education, current number of children, attending religious service, and media exposure (radio and television). We also included questions on ideational factors (contraception awareness, self-efficacy, rejection of myth and rumor, intra-family discussion and family support), pre-coded with binary or 5-point likert scale responses. Because we included both married and nonmarried women, intention to use a modern contraception broadly defined as intent to use any modern contraceptive at any point in the future.

Trained enumerators collected the data using tablets programmed with Open Data Kit, an open-source application, on FP indicators from households. The original questionnaire was developed in English and then translated into the local languages (Afar, Amharic, Oromiffa, Gambela, and Somali) and back translated to English to check for consistency. The software and tablets allowed for automatic uploads to a centralized data storage system. When instantaneous data submission was not possible (owing to poor connectivity), data were saved and uploaded once internet connectivity was reestablished.

### Data analysis

We analyzed the data using Statistical Package for the Social Sciences (SPSS) version 20. For each region, we calculated descriptive measures that characterize the study population and estimated the prevalence of intention to use FP for each region (Table 1).

**Table 1:** Summary of the sociodemographic variables

Background characteristics	Study region				
	Afar (n = 643)	Benishangul-Gumuz (n = 794)	Gambela (n = 752)	Somali (n = 702)	Total (n = 2891)
Residence					
Urban	175 (27.2)	142 (17.9)	224 (29.8)	215 (30.6)	756 (26.2)
Rural	468 (72.8)	652 (82.1)	528 (70.2)	487 (69.4)	2135 (73.8)
Education					
No education	504 (78.4)	305 (38.4)	219 (29.1)	497 (70.8)	1525 (52.7)
Primary	107 (16.6)	318 (40.1)	279 (37.1)	95 (13.5)	799 (27.6)
Secondary	17 (2.6)	93 (11.7)	178 (23.7)	72 (10.3)	360 (12.5)
Above secondary	15 (2.3)	78 (9.8)	76 (10.1)	38 (5.4)	207 (7.2)
Average age, in years (SD)	27.2 (6.66)	27.5 (8.17)	25.04 (7.24)	27.16 (7.30)	26.85 (7.50)
Partner’s education					
No education	414 (64.4)	207 (26.1)	170 (22.6)	359 (51.1)	1150 (39.8)
Primary	77 (12)	238 (30)	113 (15)	36 (5.1)	464 (16.1)
Secondary	27 (4.2)	97 (12.2)	125 (16.6)	57 (8.1)	306 (10.6)
Above secondary	28 (4.4)	94 (11.8)	181 (24.1)	53 (7.5)	356 (12.3)
Not applicable	97 (15.1)	158 (19.9)	163 (21.7)	197 (28.1)	615 (21.3)
Marital status					
Never married	142 (22.1)	102 (12.8)	140 (18.6)	148 (21.1)	532 (18.4)
Married/cohabited	478 (74.3)	636 (80.1)	591 (78.6)	506 (72.1)	2211 (76.5)
Divorced//widowed	23 (3.6)	56 (7.1)	21 (2.8)	48 (6.8)	148 (5.1)
Exposure to radio or TV					
Yes	253 (39.3)	322 (40.6)	152 (20.2)	250 (35.6)	977 (33.8)
No	390 (60.7)	472 (59.4)	600 (79.8)	452 (64.4)	1914 (66.2)
Family income					
Lowest	154 (24.0)	159 (20.0)	156 (20.7)	227 (32.3)	696 (24.1)
Second	109 (17.0)	194 (24.4)	148 (19.7)	110 (15.7)	561 (19.4)
Middle	137 (21.3)	185 (23.3)	198 (26.3)	98 (14.0)	618 (21.4)
Fourth	122 (19.0)	101 (12.7)	112 (14.9)	138 (19.7)	473 (16.4)
Higher	121 (18.8)	155 (19.5)	138 (18.4)	129 (18.4)	543 (18.8)
Religion					
Muslim	622 (96.7)	387 (48.7)	26 (3.5)	678 (96.6)	1713 (59.3)
Protestant Orthodox Catholic Other	2 (0.3) 19 (3) 0 (0) 0 (0)	106 (13.4) 276 (34.8) 5 (0.6) 20 (2.5)	598 (79.5) 80 (10.6) 14 (1.9) 34 (4.5)	2 (0.3) 21 (3) 1 (.1) 0 (0)	708 (24.5) 396 (13.7) 20 (0.7) 54 (1.9)
Attend religious services					
At least once a day	323 (50.2)	93 (11.7)	240 (31.9)	104 (14.8)	760 (26.3)
At least once a week	167 (25.9)	557 (70.2)	443 (58.9)	385 (54.8)	1552 (53.7)
At least once a month	59 (9.2)	30 (3.8)	55 (7.3)	111 (15.8)	255 (8.8)
Never/few times a year	94 (14.6)	114 (14.4)	14 (1.9)	102 (14.5)	324 (11.2)
Employment status					
Housewife Pastoralist Farmer Student House maid Private business Employed Daily laborer	450 (70.2) 153 (23.9) 12 (1.9) 36 (5.6) 0 (0.0) 28 (4.4) 30 (4.7) 2 (0.3)	324 (41.0) 8 (1.0) 320 (40.5) 112 (14.2) 4 (0.5) 55 (7.0) 51 (6.5) 2 (0.3)	393 (52.6) 3 (0.4) 52 (7.0) 281 (37.6) 4 (0.5) 48 (6.4) 82 (11) 7 (0.9)	478 (68.8) 39 (5.6) 12 (1.7) 74 (10.6) 5 (0.7) 56 (8.1) 36 (5.1) 11 (1.6)	1645 (51.9) 203 (6.4) 396 (12.5) 503 (15.9) 13 (0.4) 187 (5.9) 199 (6.3)) 22 (0.7)
Prior contraceptive use					
Yes No	109 (16.9) 534 (83.1)	517 (65.1) 277 (34.9)	272 (36.2) 480 (63.8)	111 (15.8) 591 (84.2)	1009 (34.9) 1882 (65.1)
Intend to use					
contraceptives Yes No	140 (21.8) 503 (78.2)	595 (74.9) 199 (25.1)	377 (50.1) 375 (49.9)	141 (20.1) 561 (79.9)	1253 (43.3) 1638 (56.7)

Additionally, we used the 41 ideation-measuring questions to explore the effect of personal ideation on intention to use contraceptives. For the 41 ideation measures, we converted all responses to binary form (0, 1) to generate an ideation score using a simple additive index to determine each participant women score out of a total of 41 points. In the conversion process, favorable responses/attitudes/perceptions were given 1 and the less favorable ones, including neutrals, were given 0. We further grouped these 41 items into five ideational factors based on combination of interrelated ideation measuring variables through checking their reliability and inter-consistencies using confirmatory factor analysis (CFA), according to Babalola [19] and using SPSS’s Analysis of a Moment Structures version 20.

The five ideation factors were contraception awareness (composed of 12 ideation items), self-efficacy (7 items), rejection of myth and rumor (10 items) intra-family discussion (6 items), and family support (6 items). Contraception awareness and rejection of myth and rumor fall under the cognitive domain; self-efficacy belongs to the emotional domain; and intra-family discussion and family support are under the social domain of the ideation model we employed.

The overall acceptance of the structured hypothesis was decided using goodness-of-fit measures, reliability measures, and estimates of the standardized factor loadings. Reliability (Cronbach’s alpha, α) was used to assess the degree to which the items capturing the same factor of interest are homogeneous. Alpha values of 0.70 or greater indicated adequate internal consistency. Standardized factor loading values (λ) were used to decide whether items are reflective of or a best indicator of the respective factors. A standardized loadings value of 0.60 or greater for reflective items was considered acceptable.

Upon completion of the CFA, we conducted a simple binary logistic regression analysis to select potential candidate factors; factors with *p*-value ≤ 0.25 were considered for the multiple binary logistic regressions. Finally, we conducted a multiple logistic regression to assess the effect of different demographic and ideational factors variables on the intent to use contraceptives. Factors with a *p*-value of < 0.05 were considered statistically significant. All the analyses, including the CFA, were conducted independently for each region.

### Ethical considerations

Ethical clearance was obtained from the institutional review board of Saint Paul’s Hospital Millennium Medical College (SPHMMC) on July 9, 2016, with a reference number P.M/23/29/2016. Individual verbal informed consent was obtained before proceeding to the data collection. All information obtained from the individual subjects was kept confidential. Coding and aggregate reporting were used to eliminate respondents’ identification and ensure anonymity.

## Results

### Sociodemographic characteristics of study participants

A total of 2891 WRA participated across the four emerging regions of Ethiopia (see Table 1). Participants from Gambela were slightly younger (25 years old) on average than those from Afar, Benishangul-Gumuz, and Somali, who averaged around 27 years old. The majority of the participants in all four regions reside in rural areas and were married or cohabitating. A greater proportion of women from Afar and Somali regions had no formal education (more than two-thirds of the participants, compared with less than 40% in Benishangul-Gumuz and Gambela) and did not work outside of the house. Almost all participants in Afar and Somali region were Muslim, the majority in Gambela were Protestant, and Benishangul-Gumuz had the religious greatest heterogeneity.

Among the study participants, Afar and Somali had low rates of previous contraceptive use (between 15.8% and 16.9%). Slightly more than one-third of women from Gambela and 65% of women from Benishangul-Gumuz reported previously using a modern contraceptive method.

### Ideation score versus intent to use contraceptives in the four emerging regions

**Fig. 1:**
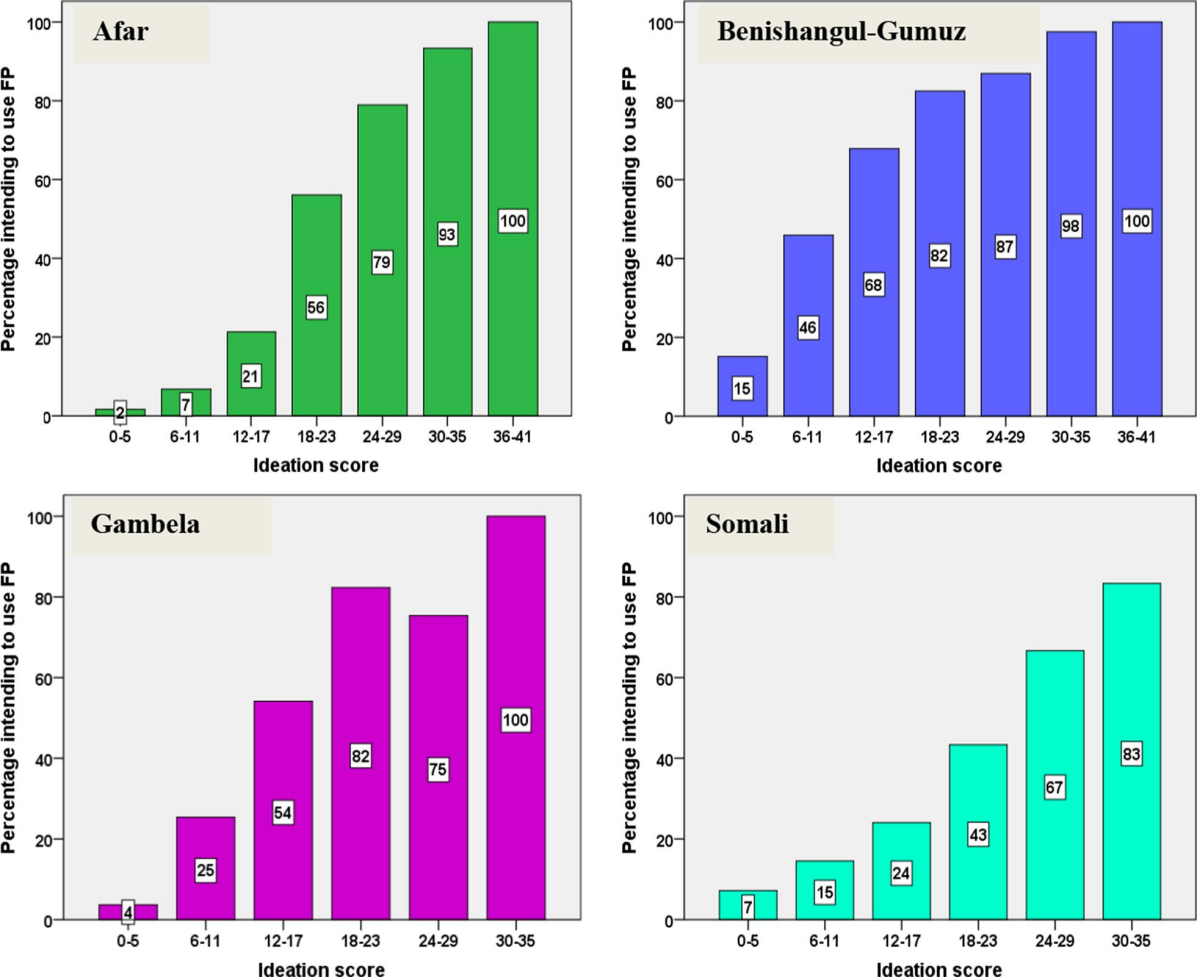
Percentage of women intending to use FP by ideation score, by region. ideation score is calculated out of 41 (representing the 41 ideation items) for each women; the maximum score in Afar, Benishangul-Gumuz, Gambela, and Somali is 37, 41, 34, and 35 respectively. Percentage intending to use FP represents the percentage of women in specific ideation score category intending to use FP relative to the total number of women in that specific ideation score category

The proportion of women intending to use contraceptives is higher (74.9%) in Benishangul-Gumuz, whereas it is comparable in Afar and Somali (21.8% and 20.1%, respectively; Table 1). The ideation score of these 41 items has a low mean (standard deviation). In all study regions, the proportion of women intending to use contraceptives in the future increases when their ideation score increases (Fig. 1).

### Confirmatory factor analysis

Based on the results of CFA, the hypothesized five-ideation factor CFA model fits the sample data reasonably. Even though all factor loadings are significant (*p* < 0.05), some of the items have low (< 0.60) standardized loadings (represented by “-” in Table 2) on factors, suggesting that they are unreliable indicator of their respective factors (Table 2). Therefore, for each region, the five ideational factors were generated using items with standardized factor loadings values of ≥ 0.60 in that particular region.

**Table 2:** Findings of CFA; standardized factor loadings and factors reliability measure in the four emerging regions

		Afar		Benishangul-Gumuz		Gambela		Somali	
No.	Factor/items	α	λ	α	λ	α	λ	α	λ
	Contraception awareness	0.87		0.85		0.90		0.87	
1	Aware of female sterilization		0.87		0.61		–		–
2	Aware of male sterilization		0.84		–		–		–
3	Aware of IUD		0.77		–		–		0.62
4	Aware of injectables		–		–		0.87		0.64
5	Aware of implants		–		–		0.80		0.79
6	Aware of pill		–		–		0.85		0.67
7	Aware of male condom		–		0.61		0.93		0.77
8	Aware of female condom		0.68		0.65		0.69		–
9	Aware of lactation amenorrhea		–		–		–		–
10	Aware of rhythm		–		0.66		–		0.63
11	Aware of withdrawal		0.71		0.65		–		–
12	Aware of emergency contraceptive		0.72		0.64		–		0.61
	Self-efficacy	0.90		0.89		0.95		0.89	
1	Perceived self-efficacy for starting a conversation with partner about FP		0.67		0.78		0.79		0.78
2	Perceived self-efficacy for convincing partner that they should use a FP method		0.77		0.78		0.85		0.80
3	Perceived self-efficacy for obtaining a FP method if decided to use one		0.78		0.89		0.92		0.89
4	Perceived self-efficacy for using a FP method even if partner doesn't want you to		0.69		–		0.82		–
5	Perceived self-efficacy for using a FP method even if no friend or neighbor uses		0.84		0.87		0.95		0.77
6	Perceived self-efficacy for using a FP method even if religious leader did not think she should		0.69		0.73		0.77		–
7	Perceived self-efficacy for getting to a place where contraceptives are provided if needed		0.77		0.70		0.89		0.71
	Rejection of myth and rumor	0.94		0.86		0.90		0.92	
1	Disagreed that use of contraceptive injection can make a woman sterile		0.70		–		–		0.77
2	Disagreed that people who use contraception end up with health problems		0.79		–		0.80		0.81
3	Disagreed that contraceptives can harm your womb		0.83		0.74		0.85		0.85
4	Disagreed that contraceptives reduce women's sexual urge		0.85		0.68		0.82		0.71
5	Disagreed that contraceptives can cause cancer		0.83		0.78		0.87		0.80
6	Disagreed that contraceptives can give you deformed babies		–		–		0.87		0.70
7	Disagreed that contraceptives are dangerous to your health		–		–		0.72		0.81
8	Disagreed that women who use FP may become promiscuous		–		–		–		0.64
9	Disagreed that women who cook cannot use FP		–		–		0.71		0.68
10	Disagreed that women who do not get enough nutrition should not use FP		0.61		–		0.64		–
	Intra-family discussion	0.84		0.77		0.82		0.74	
1	Discussed FP with mother		0.65		–		0.77		–
2	Discussed FP with mother-in-law		0.68		0.68		–		0.78
3	Discussed FP with aunt		0.74		–		0.72		–
4	Discussed FP with sister		0.73		–		–		–
5	Discussed FP with sister-in-law		0.70		0.71		0.61		0.77
6	Discussed FP with father		0.65		–		0.69		–
	Family support	0.88		0.86		0.91		0.83	
1	Perceived that mother would support my use of contraceptives		–		0.67		0.70		–
2	Perceived that mother-in-law would support my use of contraceptives		0.82		0.88		0.90		0.86
3	Perceived that sister-in-law would support my use of contraceptives		0.88		0.82		0.89		0.88
4	Perceived that father would support my use of contraceptives		0.85		0.66		0.79		0.63
5	Perceived that father-in-law would support my use of contraceptives		0.76		0.78		0.89		0.87
6	Perceived that religious leader would support my use of contraceptives		–		–		–		–

### Multiple binary logistic regressions: association of different demographic and ideational factors with the intent to use contraceptives

Results of the multiple binary logistic regression for potential candidates selected using the binary logistic regression is presented for each region in Table 3.

**Table 3:** Association of ideation and different demographic factors with intention to use contraceptives, multiple binary logistic regression

Variable	Afar Benishangul-Gumuz		Gambela Somali	
	AOR	AOR	AOR	AOR
Residence				
Rural (comparison group)				
Urban	3.08*	0.87	0.59	NS
Education				
No education(comparison group)				
Primary Secondary Above secondary	1.22* 1.15 3.58*	1.79* 5.06* 1.29	2.26 1.50 0.78	1.48 0.77 1.03
Age (years)				
15–24 (comparison group)				
25–34 35–49	0.76 0.54	0.49* 0.13*	0.97 0.36*	0.69 0.29*
Partner’s education				
No education (comparison group)				
Primary Secondary Above secondary	0.56 0.93 0.71	1.63 2.73 2.50	0.75 1.09 0.87	0.31 0.63 0.70
Marital status				
Never married (comparison group)				
Married/cohabited Divorced/widowed	NS	0.59 0.60	NS	NS
Exposure to radio or TV				
No (comparison group)				
Yes	1.75	0.80	1.19	1.50
Family income				
Lowest (comparison group)				
Second Middle Fourth Higher	NS	NS	0.48 0.39 0.47 0.63	1.52 1.53 1.17 0.83
Religion				
Muslim (comparison group)				
Non-Muslim	3.28	NS	1.21	3.33
Attend on religious services				
At least once a day (comparison group)				
At least once a week At least once a month Never/few times a year	1.23 0.88 0.57	1.91 2.19 2.79*	1.85* 1.15 1.86	2.03 1.37 1.61
Employment status				
House wife(comparison group)				
Pastoralist Farmer Employed Other	NS	0.97 0.92 0.66 0.50	NS	0.26 0.24 0.61 0.47
Prior contraceptive use				
Yes (comparison group)				
No	0.29*	0.59	0.18*	0.07*
No. of children				
Zero (comparison group)				
1–2 3–4 $\ge 5$	0.43 0.32* 0.29*	3.07* 3.13* 6.24*	0.32* 0.31* 0.15*	1.79 1.35 2.24
Ideational factors				
Contraception awareness	6.06*	1.22	1.88	4.22
Self-efficacy	3.85*	4.76*	6.25*	3.45*
Rejection of myth and rumor	2.34*	2.83*	2.0*	1.13
Intra-family discussion	1.42	0.48	0.49	1.66
Family support	1.54*	0.96	1.28	1.39

*NS* not selected by the simple binary logistic regression, *AOR* adjusted odds ratio

*p-value < 0.05

### Afar region

The demographic factors (residence, education, prior contraceptive use, and number of children) and four ideational factors (contraception awareness, self-efficacy, rejection of myth and rumor and family support) showed significant association with the intent to use contraceptives at 5% level of significance.

Women who had better awareness of contraceptives had a 6.06 times higher chance of intending to use contraceptives compared to women with a low awareness of contraceptives. Increased family support was associated with a 54% higher likelihood of the intent to use contraceptives (Table 3).

### Benishangul-Gumuz region

The demographic factors (education, age, attend on religious services and number of children) and the ideational factors of self-efficacy and rejection of myth and rumor showed significant association with the intent to use contraceptives at the 5% level of significance.

Women who had high rejection of myth and rumor had a 2.83 times higher chance of intending to use contraceptives compared to lesser scores on rejecting myth and rumors. High self-efficacy was associated with a 4.76 times higher chance of intending to use contraceptives (Table 3).

### Gambela region

Demographic factors (age, attend on religious services, prior contraceptive use, and number of children) and two ideational factors (self-efficacy and rejection of myth and rumor) showed significant association with the intent to use contraceptives at 5% level of significance.

Women who had high levels of rejection of FP myth and rumor were 2.0 times more likely to report intention of future contraceptive use, compared to women with lesser scores on rejection of myths and rumors. High self-efficacy in FP was associated with a 6.25 times higher level of reporting an intention of future contraceptive use (Table 3).

### Somali region

The demographic factors of age and prior contraceptive use and only one ideational factor of self-efficacy in FP showed significant association with the intent to use contraceptives at 5% level of significance. High self-efficacy was associated with a 3.45 times higher chance of intending to use contraceptives (Table 3).

## Discussion

This study revealed the differences and commonalities of ideational factors as determinants of contraceptive use intentions in the four emerging regions of Ethiopia. The findings showed that for each region, higher contraceptive ideation scores were associated with greater intention of future contraceptive use. This finding is consistent with previous findings suggesting that higher contraceptive ideation is one important means to promote intentions to use contraceptives [8, 18–20]. Even with differences in contraceptive prevalence and reproductive health outcomes across the four emerging regions of Ethiopia, a similar positive correlation of contraceptive ideation and the intent to use contraceptives was observed in each of the regions.

We observed variation across regions on the effect of ideational factors on intention to use contraception. Of the five dimensions of ideation, self-efficacy was an important predictor of the intention to use contraception in all regions of the study, a result consistent with evidence from prior research [19, 20]. Rejection of myth and rumor was the other important dimension of ideation in all regions except in Somali. Contraception awareness and family support were significant in Afar region only. In Afar household decision making, men are generally the authority figure and have the final say [21]. This might be the reason for the strong influence of family support as the strong determinant of the intent to use FP.

The role that the different ideation dimensions play in contraceptive use intention can help guide health education initiatives. The results from this study provide information to regional/district health offices to tailor their areas of focus to improve future contraceptive use. Self-efficacy was found to be a significant predictor variable on the intent to use contraceptives in all the four regions. Thus, the communication programs in the four regions should focus on increasing self-efficacy for contraceptive use. Strategies for strengthening self-efficacy for contraceptive use should include encouraging clients to use contraceptives for the first time and develop mastery of the practice. Along with communications emphasizing self-efficacy, regional/district health offices should identify and address psychological, logistic, and structural barriers to contraceptive access. Family planning programs should provide opportunities for women to learn and practice how to communicate with their spouses about contraceptive use. Other relevant strategies should include opportunities for the audience to learn from a satisfied contraceptive user similar in other respects to the non-using audience. Observing relevant behaviors in others allows one to form “a conception of how new behavior patterns are performed, and on later occasions the symbolic construction serves as a guide for action” [22]. Modeling relevant behaviors can be implemented in small groups or through the mass media. Promoting discussion about contraceptive use with significant others and encouraging personal advocacy in favor of contraceptive use among the intended audience are also important to build self-efficacy.

The rejection of myth and rumor dimension of ideation was found to be a significant predictor variable on the intent to use contraceptives in Afar, Benishangul-Gumuz, and Gambela. For these regions, effectively demystifying myths and rumors about contraceptives through strategically designed messages that provide factual information on contraceptives will be vital to improve the intention to use contraceptives. Mass media and community conversations that allow participants to discuss the prevailing myths about contraceptives in their community and critically examine their personal beliefs about contraceptives may be helpful. Given the important role of family support in Afar, the relevant regional/district health office should broaden the reach of family planning education programs to include in-laws—particularly mothers-in-law—to shift ideation of contraceptive use.

In addition to the ideation factors, we found other sociodemographic variables that predicted the intent to use contraceptives and that aligned with previous findings in the literature. In the Afar and Benishangul-Gumuz regions, education was found to be a positive predictor on intention to use contraceptives. Women’s educational level on contraceptive use has been well documented, especially in developing countries where high proportions of women are not using FP services [19, 23–25]. However, we did not see the same effect in Gambela and Somali region, perhaps because everyone (educated or not) adheres to decrees from ethnic/religious leaders.

Younger women had higher intentions to use contraceptives in the future. Studies in low and middle income countries [19, 23] reveal that younger women are more likely to use FP services than older women. This difference may be due to continuous education attainment opportunities that might improve a younger generation’s knowledge about utilizing contraception.

In this study, women’s place of residence affects contraception use intention. In particular, women belonging to a pastoralist community had lower intentions of using contraceptives compared with non-pastoralist ones, consistent with other studies [24, 26]. Pastoralist communities of Ethiopia, which are hard to reach and mobile, have comparatively less access to and provision of health care services such as medicines, health equipment, health providers, and other services. These factors combine to result in low quality, high cost services; coupled with the lack of transportation, these factors result in unmet demand for health services, including family planning.

Parity had an uneven effect. In Afar and Gambela regions, women who had a greater number of children expressed less the intent to use contraceptives, whereas the opposite was true for Benishangul-Gumuz, where women’s intention to use contraceptives increased as the number of children increased. Similar research from Bangladesh [23] showed that women with large number of children tended to use contraceptives more compared to women with fewer children.

This study has limitations. The information collected is self-reported. There is, therefore, the possibility that the ideation and intention to use family planning responses may have been affected by social desirability bias. Prior to data collection, the data collectors were trained to minimize subjectivity and the influence of social desirability. Only selected districts were included in the study raising the question of generalizability; this may not be an important issue in the Somali and Afar regions, which have a homogeneous ethnic and religious distribution. For the Benishangul-Gumuz and Gambela regions, however, where there is ethnic diversity, we attempted to include districts from each ethnic group.

## Data Availability

The dataset supporting the conclusions of this article is included within the article (and its Additional file). The raw data used in this study are available from the corresponding author on reasonable request.

## Abbreviations

AOR: Adjusted odds ratio
CFA: Confirmatory factor analysis
CPR: Contraceptive prevalence rate
EDHS: Ethiopian Demographic and Health Survey
FMoH: Federal Ministry of Health
FP: Family planning
SPHMMC: Saint Paul’s Hospital Millennium Medical College
SPSS: Statistical Package for the Social Sciences
WRA: Women of reproductive age
